# Efficacy of Praziquantel Treatment and *Schistosoma Mansoni* Infection among Primary School Children in Kemisse Town, Northeast Ethiopia

**DOI:** 10.4314/ejhs.v32i3.20

**Published:** 2022-05

**Authors:** Meslo Sema Berhanu, Seyfe Asrade Atnafie, Tahir Eyayu Ali, Aderaw Adamu Chekol, Habtamu Biazin Kebede

**Affiliations:** 1 Department of Medical Laboratory Sciences, College of Medicine and Health Sciences, Debre Tabor University, Debre Tabor, Ethiopia; 2 Department of Pharmacology, College of Medicine and Health Sciences, University of Gondar, Gondar, Ethiopia; 3 Department of Clinical Laboratory Science, College of Medicine & Health Sciences, Wollo University, Dessie, Ethiopia; 4 Department of Microbiology, Immunology and Parasitology, College of Health Sciences, Addis Ababa University, Addis Ababa, Ethiopia

**Keywords:** Schistosoma mansoni, Efficacy, Praziquantel, Cure rate, Egg reduction rate

## Abstract

**Background:**

Schistosoma mansoni infection is endemic in Ethiopia. The epidemiology of S. mansoni and the efficacy of praziquantel among schoolchildren have not been well documented in different parts of the country including our study area. Therefore, this study aimed to determine the status of S. mansoni infection and evaluate the therapeutic efficacy of praziquantel among school children in northeast Ethiopia.

**Methods:**

A comparative cross-sectional study was conducted among 499 children of two preschool children. Stool specimens were collected and microscopically examined using Kato-Katz (41.7 gram) methods. Positive children were treated with a single oral dose of praziquantel at 40 mg/kg body weight. Egg reduction and cure rates were assessed 4 weeks post-treatment to evaluate the therapeutic efficacy of praziquantel against S. mansoni infection.

**Results:**

The overall prevalence of S. mansoni infection among the schoolchildren was 52.1% with a mean intensity of 546 eggs per gram of stool. Majorities of the S. mansoni infections were moderate to heavy intensity, with only 5.0% light infections. Praziquantel administered at a single oral dose of 40 mg/kg achieved a cure rate of 91.7% and reduced the egg rate by 86.8%. Twenty-one schoolchildren remained infected at 4 weeks post-treatment, among which 6 and 15 children had moderate and light infections, respectively.

**Conclusions:**

S. mansoni prevalence among primary school children in Northeast Ethiopia was high, highlighting the need to implement school-based chemotherapy with annual frequency. The efficacy of praziquantel at 40 mg/kg is sufficient to permit continued use in treating S. mansoni-infected schoolchildren.

## Introduction

Schistosomiasis, a water-borne infection caused by *Schistosoma* trematode worms, is one of the most prevalent parasitic diseases in the world. It is endemic in 78 countries and continues to be a global public health and socio-economic concern in the developing world. More than 700 million people worldwide are at risk of infection with schistosomiasis, and about 250 million people are currently infected. An estimated 90% of all cases, and most of the severely affected or seriously ill cases, are now concentrated in Sub-Saharan Africa (SSA) where few control efforts are made (1,2). Moreover, the number of deaths involving schistosomiasis in SSA is estimated to be higher than in any other region of the world with as high as 200,000 deaths per year (2). Climate changes and global warming, proximity to water bodies, irrigation and dam construction, occupational activities such as fishing and farming, and poverty have been often linked with an increase in transmission of the disease in SSA countries (3,4).

Humans are usually infected by five species of Schistosoma, but the two most important species causing schistosomiasis in SSA countries including Ethiopia are *Schistosoma mansoni* and *S. haematobium* (3,5). *S. mansoni* causes intestinal schistosomiasis and it is the chief cause of clinical abnormalities such as hepatomegaly, splenomegaly, and periportal fibrosis in various SSA countries (3,6). School-aged children harbour the highest prevalence and intensity of infections in SSA, as the hygienic and playing behaviour in infected water bodies increases the risk of being infected by *S. mansoni* (2,7). Previous studies of schistosomiasis-infected children in SSA revealed that schistosomiasis due to *S. mansoni* can cause growth stunting, fatigue, weakness, impairment of memory and cognitive reasoning, and increased risk of anaemia that can also contribute to reduced cognitive function, school absenteeism and higher drop-out rates (8–10). These infections are also reported to increase susceptibility to co-infections and influence the severity of other infectious diseases such as malaria in school children (11). Failure to treat schoolchildren, therefore, significantly affects the quality of life of infected children and potentially contributes to the socioeconomic burden in disease-endemic areas.

Currently, the anti schistosomal drug of choice for the treatment of schistosomiasis is praziquantel (PZQ), which is effective in reducing disease-associated morbidity (12–14). It is highly effective against all *Schistosoma species* that are known to infect humans and is the least expensive, easiest to use, and most readily available of all currently available schistosomicides (15). It is recommended that school-aged children and high-risk groups of adults in communities with a prevalence of 10% to 50% use it once every two years or once a year when the prevalence is above 50% and once every three years when the prevalence does not exceed 10% (16). While chemotherapy with PZQ is considered the mainstay of schistosomiasis control, there is a growing concern regarding the low cure rate and drug resistance. The problem of reduced therapeutic efficacy has been also reported from different geographic locations, indicating the need for constant monitoring of PZQ efficacy in endemic areas.

In Ethiopia, both urinary and intestinal forms of schistosomiasis occur although the latter is the most widespread and highly prevalent (17). Several epidemiological studies indicate that intestinal schistosomiasis caused by *S. mansoni* is highly endemic in different parts of Ethiopia with prevalences as high as 90% in school-aged children (18). Despite this, the epidemiology of *S. mansoni* and the therapeutic efficacy of PZQ among schoolchildren have not been well documented in different parts of the country including our study area. Therefore, this study was conducted to determine the prevalence and intensity of *S. mansoni* infections and evaluate the therapeutic efficacy of PZQ at 40 mg/kg among primary school children of Kemisse Town, northeast Ethiopia.

## Methods

**Study area and population**: This study was conducted among 499 primary school children of Kemisse Town, a small town in “Oromia Special Zone” in Amhara Regional State, Northeast Ethiopia. The town is located approximately 350 km northeast of the capital Addis Ababa at a latitude and longitude of 10°43′0″N 39°52′0″E. The area has an elevation of 1,424m above sea level with an average temperature and rainfall of 19.36°C and 950 mm, respectively. More than 90 % of the population in the area is composed of the Oromo ethnic group, and agriculture and trade are the main income source for the majority of the community. There are Worke and Kachuri Rivers, which cross in the town and the community use these rivers for different domestic purpose well schoolchildren have a contact at different sites while passing through the town. The two primary schools, namely Kachuri and Haromssa in which the parasitological survey was conducted, are situated close to the rivers. These predispose the children to water-borne diseases during playing, washing, and swimming in the water bodies. Children attending primary schools during the study period who looked healthy were included in the study. Schoolchildren who were treated with any anthelmintic drug in the past three months were excluded from the study.

**Study design**: A comparative cross-sectional study design was employed among school-aged children of two purposively selected primary schools of Kemisse Town near rivers and streams/swamps suspected as sources of schistosomiasis in the area. Purposive sampling of the schools was done based on schistosomiasis case information obtained from the health centers in the town where the target primary schools are located based on the World Health Organization (WHO) guidelines for assessing the efficacy of anti-helminthic drugs against schistosomiasis and soil-transmitted helminthiases (18).

**Sample collection and processing**: A structured questionnaire was used to gather relevant information from each student. Approximately 5 g of fresh stool specimens were collected from each child and processed using the Kato-Katz (two slides per individual) at baseline and one month after PZQ treatment. Only children who were infected with *S. mansoni* at baseline and treated with praziquantel were re-examined one month after treatments. The Kato-Katz thick smear prepared for each child was examined for both the detection and the quantification of *S. mansoni* eggs and the presence of other intestinal helminths. The study utilized the examination of Kato-Katz slides (42.7 mg stool/slide) for each individual (19). Slides were prepared and examined by two experienced laboratory technicians within an hour.

The number of *S. mansoni* eggs was counted and was converted into eggs per gram of stool (EPG). The intensity of infection was calculated as the arithmetic mean of 2 slides per child and classified into light (1–99 EPG), moderate (100–399 EPG), or heavy (≥ 400 EPG) according to WHO guidelines (20). For quality control, 10% of the slides were randomly selected and re-examined quantitatively by two independent medical laboratory experts who were blinded to the primary result.

**Praziquantel treatment**: All children who were positive for helminth infections were treated with appropriate anthelminthic drugs, but only the children treated with a single oral dose (40 mg/kg) of PZQ were taken on the fourth week to monitor the cure and egg reduction rates. The drug was administered with a glass of water following the confirmation that the child ate at home or ate food that was provided by the investigating team. Nurses who followed students who were treated with PZQ for four hours and those who vomited within two hours after oral administration of the drug were excluded from the analysis.

**Statistical analysis**: Data were entered into “EpiData v4.6” and were exported into Software Package for Social Sciences (SPSS) version 23.0 software for analysis. Descriptive statistics were used to summarize the collected data. Chi-square (x_2_) test and one-way ANOVA were used to test for differences in the prevalence of infections and arithmetic mean of egg excretion, respectively. P-values < 0.05 were considered statistically significant. The cure rate (CR) was calculated as [the number of children excreting no *S. mansoni* eggs after treatment /the number of children who had eggs in their stool before treatment] x 100. Egg reduction rate (ERR) was calculated as [1 − (arithmetic mean EPG after treatment/ arithmetic mean EPG before treatment)] x 100 (20).

**Ethical considerations**: The protocol of this study was reviewed and approved by the Ethical Review Committee of the College of Medicine and Health Science of Wollo University. Permissions were obtained from Oromia Special Zone Health Department, Kemisse District Health, and Educational Bureaus to conduct the study. The objective of the study was explained to the school administration, primary health care providers, and parents/guardians of the children. Written informed consent for the children was obtained from each parent/guardian. Experienced nurses treated children who were found positive for helminth infections with appropriate anthelminthic drugs. Schoolchildren who vomited within two hours of PZQ administration were referred to the nearby Health Center for further treatment.

## Results

**Socio-demographic characteristics of study subjects**: Four hundred and ninety-nine schoolchildren (263 males and 236 females) were included in the study. The age range of interviewed children was 5 to 18 years with a median of 11 (± 2.1 SD) years and larger proportions (74.3%) were in the age range of 10–14 years.

**Prevalence and intensity of *S. mansoni* infection**: Out of 499 study subjects, 52.1% (95% CI: 47.7–56.5) were found to be positive for *S. mansoni* infection. The prevalence of infection was highest in Kachur primary school 152(61.5 %) than Haromssa 108 (42.9) (χ^2^=8.097; *P* = 0.004). The difference might be due to the proximity of the rivers and the presence of a swamp close to the school, which forces children to spend most of their time playing in water contaminated with an infective stage of *S. mansoni*. Boys were more infected (57.4%) compared to girls (46.2%; *χ*^2^ = 6.284, *P* = 0.012) and this may be due to the playing behavior of the boys in infected water bodies. The prevalence of infection was 45.0% for the 5–9, 53.9%, for 10–14, and 57.9% for the 15–18 years age groups. Although the rate of infection increased as a function of age, no significant difference was detected between the age groups (*χ*^2^ = 2.829, *P* = 0.243) (Table 1).

**Table 1:** Socio-demographic characteristics and prevalence of *S. mansoni* among children of two primary schools in Kemisse Town, Northeast Ethiopia, 2019

Variables	Responses	N (%)	*S. mansoni* infection status		X^2^ values	P values

			Positive n (%)	Negative n (%)		
Sex	Boys	263 (52.7)	151 (57.4)	112 (42.6)	6.284	0.012
	Girls	236 (47.3)	109 (46.2)	127 (53.8)		
Age	5–9	109 (21.8)	49 (45.0)	60 (55.0)	2.829	0.243
group,	10–14	371 (74.3)	200 (53.9)	171 (46.1)		
years	15–18	19 (3.8)	11 (57.9)	8 (42.1)		
School	Kachur	247 (49.5)	152 (61.5)	95 (38.5)	8.097	<0.005
	Haromssa	252 (50.5)	108 (42.9)	144 (57.1)		
Total n (%)		499	260 (52.1)	239 (47.9)		

The overall mean number of eggs excreted per gram of stool (EPG) of *S. mansoni* infected children was 546.1, ranging from 24 to 4704 EPG. The intensity of infection varied by school, with the highest 642 EPG in Kachur and 411 EPG in Haromssa primary school (*P* = 0.005). This could also be due to the proximity of transmission sites to schools, hence frequent exposure to *S. mansoni* infection. The intensity of *S. mansoni* infection was higher among males (609 EPG) than females (459 EPG) and the difference was statistically significant (*P* = 0.039). The intensity of infection among the infected students increased significantly with each age category, from 493 EPG for the age group 5–9 years to 1041 EPG for age 15–18 years (*P* = 0.014) (Table 2). This could be due to the repeated exposure of males and higher age groups to *S. mansoni* infection due to their playing habits. Majorities of the *S. mansoni* infections were moderate to heavy intensity, with only 5.0% light intensity.

**Table 2:** Intensity of *S. mansoni* infection in children of two primary schools in Kemisse Town, Northeast Ethiopia, 2019

Variables		Mean EPG	Level of infection intensity			P- value

			Light, n (%)	Moderate, n (%)	Heavy, n (%)	
School	Kachur	642	3 (1.2)	75 (28.9)	74 (28.4)	0.005
	Haromessa	412	10 (3.8)	50 (19.2)	48 (18.5)	
Sex	Boys	609	6 (2.3)	71 (27.3)	74 (28.4)	0.039
	Girls	459	7 (2.7)	54 (20.8)	48 (18.5)	
Age group in	5–9	494	5 (1.9)	25 (9.6)	19 (7.3)	0.014
yrs.	10–14	532	8 (3.1)	95 (36.6)	97 (37.3)	
	15–18	1041	0 (0.0)	5 (1.9)	6 (2.3)	
**Overall intensity**		546	13 (5.0)	125 (48.1)	122 (46.9)	

Of the total 122 (46.9%) heavy infections of *S. mansoni,* 74 (60.7%) were in Kachur primary school and 48 (39.3%) in Haromssa. The proportion of males (28.4%) that had a heavy infection was greater than females (18.5%). The highest heavy infections due to *S. mansoni* 97 (79.5%) were observed in children aged 10–14 years followed by 19 (17.4%) in those aged 5–9 years (Table 2).

**Therapeutic efficacy of praziquantel**: Four weeks post-treatment with 40 mg/kg PZQ, stool samples were collected from 252 children who had tested positive and been treated at baseline. Of these, 21 still excreted *S. mansoni* egg; 71.4% and 28.6% had light and moderate intensity of infections, respectively.

The overall cure rate (CR) of PZQ among *S. mansoni* infected schoolchildren was 91.7%; 91.0% among boys and 92.5% among girls. Within the age groups, the CR was 90.9% among children aged 5–9 years, 92.4% in the 10–14 years age group, and 81.8% among those aged 15–18 years. The overall pre-treatment intensity of infection for 252 infected schoolchildren was 540 EPG but fell to 73 EPG 4 weeks post-treatment. Hence, the overall egg reduction rate (ERR) was 86.8%, 84.9% among boys, and 90.2% among girls (*P* = 0.218) as tabulated in Table 3. The ERR had no significant association with the age of the schoolchildren (*P* = 0.159). The CR and ERR were not associated with infection intensity (*P* = 0.126 and *P* = 0.061), respectively (Table 3).

**Table 3:** Cure rate and Egg reduction rate of PZQ against *S. mansoni* infected children of two primary schools in Kemisse Town, Northeast Ethiopia, 2019

Variables		N (%)	Cured, n (%)	P-value	Egg reduction rate (%)	P-value
Sex	Boys	145(57.5)	132 (91.0)	0.673	84.9	0.218
	Girls	107(42.5)	99 (92.5)		90.2	
Age group (years)	5–9	44 (17.5)	40 (90.9)	0.458	85.5	0.159
	10–14	197 (78.2)	182 (92.4)		77.9	
	15–18	11 (4.4)	9 (81.8%)		98.2	
Total			231 (91.7)		86.8	

## Discussion

The overall prevalence of *S. mansoni* in the present study was (52.1%). The finding was lower than the prevalence (89.9%) reported from primary school children in the Sanja area, Amhara region (21), 74.9% in Wondo Genet, Southern Ethiopia (22), 70.47% in Senbete Town, Northeastern Ethiopia (23), 58.7% in Adwa Town, Northern Ethiopia (24), and 58.6% and 77.3% in Wolaita Zones of Southern Ethiopia (25,26). However, the prevalence of infection in the present study areas was higher than those studies reported from different parts of the country, which reported the prevalence of *S. mansoni* to range from 23.9-to 49% using a similar Kato-Katz technique (27–30). The difference in prevalence may partly be explained by variation in awareness regarding *S. mansoni* transmission or due to variation in the study design, sampling techniques, sample size, and the time of the studies.

The current finding, on *S. mansoni* infection prevalence, was also lower than those reported at 41.3% from western Kenya (31), 64.3% from Northwestern Tanzania (32), and 58.1% from western Côte d'Ivoire (33).

However, this finding was higher than the prevalence reported(47.4%) from Mwea Division, Central Kenya (34), 21% from Kisumu City, Western Kenya (35), 27.8% from Western Uganda (36), and 31.4% from Giza governorate of Egypt (37). The difference might be due to the endemicity of the parasite in the areas, the climate of the areas, and the socioeconomic status of the communities living in the study areas.

In addition, the infection intensity in the present study indicated moderate to heavy infection levels (95%), which is comparable with findings from Senbete Town, Northeastern Ethiopia (23), Wondo Genet, Southern Ethiopia (22), Sanja Town, Northwest Ethiopia (18) and Damot Woide District of Southern Ethiopia (25). However, light infection intensities were reported from Adwa Town, northern Ethiopia (24), Manna District, southwest Ethiopia (28), and from western Kenya (31), western Côte d'Ivoire (33), and Western Uganda (36). Light to moderate intensity of *S. mansoni* infection among school children was also reported from the Wolaita Zone of southern Ethiopia (27) and Giza governorate of Egypt (37). The difference may be explained by the intensity of parasite transmission in the study areas and the frequency of students' contact with water bodies infested with the infective stage.

The efficacy of praziquantel (PZQ) against *S. mansoni* determined in this study was lower than those reported from a highly endemic area of north-west Ethiopia (CR = 94% and ERR = 97%) (38) and the Manna District of southwest Ethiopia (CR = 99.1% and ERR = 99.9%) (27). This difference could be due to baseline infection intensity and duration of post-treatment (three weeks of post-treatment in the Manna District study). The presence of immature stages of the parasite might also account for some of the variations observed in the present and the previous study areas as these immature stages have a high chance to survive the treatment and mature to deposit eggs in the post-treatment periods. Higher ERR (99.51%) was also reported from a previous efficacy study carried out four weeks of post-treatment in Finchaa valley, Ethiopia (39). The high rate of *S. mansoni* infection (52.1%) and heavy infection cases (46.9%) observed in the present study could in part explain the lower efficacy of PZQ in the study area. Some previous studies from different parts of Africa also reported higher efficacy of PZQ among children including Senegal (CR = 93 % and ERR = 90 %) and Cameroon (CR = 99.5–100%) (40). The difference might be due to the intensity of infection, a brand of PZQ used post-treatment duration or re-infection, and geographical location.

The efficacy of praziquantel at 40 mg/kg in the present study areas (CR = 91.7% and ERR = 86.8%) was higher than two previous studies conducted in the country including Senbete Town of northeastern Ethiopia (CR = 82.89% and ERR = 79.46%) (23) and Wondo Genet, Southern Ethiopia (CR = 73.6 % and ERR = 68.2 % (22) with similar duration of posttreatment. Previous efficacy studies conducted in Finchaa Valley and northeast Ethiopia also reported lower parasitological CR (80.9% and 83.2%, respectively) (38,39). This difference might be due to infection intensity, the presence of immature stages of the parasite, and geographical location. The efficacy of praziquantel at 40 mg/kg among school-aged children in the present study was also higher than the findings from western Niger (CR =51.7 -55.2% and ERR = 58.8–60.2%) (41), western Côte d'Ivoire (CR = 71.6% and 79.9%) (33) and Egypt (CR = 62.5%) (37). The difference in PZQ efficacy could also be explained by variation in the intensity of infection, a brand of praziquantel used, and geographical location or due to re-infection during post-treatment periods. On the other hand, the efficacy of PZQ in treating *S. mansoni* infected school children in our study area was almost comparable to those reported from Tumuga and Waja of north Ethiopia (CR = 88.99–93.44%) (42) and the Mwea Division in Central Kenya (ERR = 92.6%) (34).

*S. mansoni* prevalence among primary school children in Kemisse town, northeast Ethiopia was high. The high prevalence of *S. mansoni* among schoolchildren confirms the endemicity of intestinal schistosomiasis due to *S. mansoni* in the study areas. Given the WHO guidelines on deworming frequency according to schistosomiasis prevalence, there is a need to implement school-based preventive chemotherapy with annual frequency. Furthermore, a single oral dose of PZQ at 40 mg/kg produced a cure rate of 91.7% and an overall egg reduction rate of 86.8% 4 weeks post-treatment. The efficacy of this drug is sufficient to permit continued use in treating *S. mansoni*-infected schoolchildren in the study area, but further monitoring of its efficacy in different epidemiological settings is required.

In conclusion, our study identified a high prevalence of *S. mansoni* infection among primary school children of Kemisse town, northeast Ethiopia. More than 52% of school-aged children in our study area were infected with *S. mansoni* and majorities (95%) had moderate to heavy infection intensities. Given the WHO guidelines on deworming frequency according to schistosomiasis prevalence, these results highlight the need to implement school-based preventive chemotherapy with annual frequency. A single oral dose of PZQ administered at 40 mg/kg body weight produced a cure rate of 91.7% and an overall egg reduction rate of 86.8% 4 weeks post-treatment. The efficacy of this drug is sufficient to permit continued use in treating *S. mansoni*-infected schoolchildren in the study area. However, further monitoring of PZQ efficacy in different epidemiological settings is required.
